# Enhancing Lifestyle Change in Cardiac Patients Through the Do CHANGE System (“Do Cardiac Health: Advanced New Generation Ecosystem”): Randomized Controlled Trial Protocol

**DOI:** 10.2196/resprot.8406

**Published:** 2018-02-08

**Authors:** Mirela Habibović, Eva Broers, Jordi Piera-Jimenez, Mart Wetzels, Idowu Ayoola, Johan Denollet, Jos Widdershoven

**Affiliations:** ^1^ Department of Medical and Clinical Psychology Tilburg University Tilburg Netherlands; ^2^ Department of Cardiology Elisabeth-TweeSteden Hospital Tilburg Netherlands; ^3^ Badalona Serveis Assistencials Badalona Spain; ^4^ Department of Industrial Design Eindhoven University of Technology Eindhoven Netherlands; ^5^ Onmi Eindhoven Netherlands

**Keywords:** cardiac health, lifestyle, behavior change, eHealth, mHealth

## Abstract

**Background:**

Promoting a healthy lifestyle (eg, physical activity, healthy diet) is crucial for the primary and secondary prevention of cardiac disease in order to decrease disease burden and mortality.

**Objective:**

The current trial aims to evaluate the effectiveness of the Do Cardiac Health: Advanced New Generation Ecosystem (Do CHANGE) service, which is developed to assist cardiac patients in adopting a healthy lifestyle and improving their quality of life.

**Methods:**

Cardiac patients (ie, people who have been diagnosed with heart failure, coronary artery disease, and/or hypertension) will be recruited at three pilot sites (Badalona Serveis Assistencials, Badalona, Spain [N=75]; Buddhist Tzu Chi Dalin General Hospital, Dalin, Taiwan [N=100] and Elisabeth-TweeSteden Hospital, Tilburg, The Netherlands [N=75]). Patients will be assisted by the Do Something Different (DSD) program to change their unhealthy habits and/or lifestyle. DSD has been developed to increase behavioral flexibility and subsequently adopt new (healthier) habits. In addition, patients’ progress will be monitored with a number of (newly developed) devices (eg, Fitbit, Beddit, COOKiT, FLUiT), which will be integrated in one application.

**Results:**

The Do CHANGE trial will provide us with new insights regarding the effectiveness of the proposed intervention in different cultural settings. In addition, it will give insight into what works for whom and why.

**Conclusions:**

The Do CHANGE service integrates new technologies into a behavior change intervention in order to change the unhealthy lifestyles of cardiac patients. The program is expected to facilitate long-term, sustainable behavioral change.

**Trial Registration:**

Clinicaltrials.gov NCT03178305; https://clinicaltrials.gov/ct2/show/NCT03178305 (Archived by WebCite at http://www.webcitation.org/6wfWHvuyU).

## Introduction

Cardiovascular diseases are the leading cause of death worldwide and a major driver of health care costs [1]. Evidence shows that a large proportion of the disease burden can be explained by behavioral factors (eg, low physical activity, unhealthy diet) [2], and that approximately 80% of heart disease, stroke, and type 2 diabetes can be prevented by attenuating or eliminating these health risk behaviors [3,4]. Hence, in their recent call for action, the American Heart Association stressed the importance of lifestyle management and called for better lifestyle counseling and the development of interventions to support health behavior change in cardiac patients [5].

Despite evidence showing that changing health behaviors improves (mental) health outcomes and lowers health care costs [5], to date, lifestyle counseling is not routinely implemented in physicians’ office [6]. More precisely, physicians provide this type of counseling in only 34% of the clinic visits [7]. One of the important reasons for this is the fact that face-to-face counseling is time-consuming.

Remote technologies offer a new delivery model for promoting healthy behaviors and are increasingly used in health care settings [8]. Although these new developments provide an excellent opportunity to deliver behavior change interventions to large groups of underserved patients, the reported effect sizes have been small [8]. Possible explanations for this are the short duration of the interventions [8], the limited number of health-related behaviors addressed within the intervention program [9], the mismatch between patients’ needs or preferences and the intervention, or the lack of sound behavior change methods adopted [10]. Previous trials within the cardiac population have demonstrated that a “one size fits all” approach does not seem to work [11]—revealing the importance of personalizing the care plan and addressing patients’ needs and preferences.

Evidence shows that the conventional way of providing education about a healthy lifestyle does not result in adopting desirable health behaviors [12]. In order to produce sustainable change in health behaviors, the Do Cardiac Health: Advanced New Generation Ecosystem (Do CHANGE) study will provide a personalized theory-based behavior intervention program for three months, creating awareness of unhealthy behaviors, addressing multiple health behaviors at the same time, and changing unhealthy habits. [13].

The Do CHANGE service aims to address cardiac patients’ unhealthy habits and change these by providing them with monitoring tools (eg, Fitbit) and increasing their behavioral flexibility. The Do Something Different (DSD) behavior change program will be provided, with the objective of disrupting the habit chains that are common in our daily living. Since people generally tend to live in accordance with their habits [14], disrupting these habits may lead to higher behavioral flexibility and eventually to behavior change [13]. The DSD program has been shown to be effective in changing health behaviors in previous studies targeting different populations [15]. The Do CHANGE service will, therefore, not only provide patients with innovative tools to support behavior change, but will also offer behavioral alternatives and carefully assess patients’ needs on using these innovative tools.

The objective of the current trial is to evaluate the effectiveness of the personalized Do CHANGE service in changing unhealthy lifestyle and improving the quality of life in cardiac patients. The Do CHANGE service will be developed and evaluated in three different countries (Spain, Taiwan, The Netherlands), in order to represent patients from different cultural backgrounds. This may contribute to a higher generalizability of the study findings.

## Methods

### Design

Do CHANGE is an international (Spain, Taiwan, The Netherlands), multicenter, randomized (intervention versus care as usual) controlled trial designed to support lifestyle change in patients with cardiac disease. By increasing patients’ behavioral flexibility and providing them with innovative devices, the objectives of enhancing lifestyle change and improving patients’ quality of life are expected to be reached. The evaluation of the Do CHANGE service delivery consists of two randomized controlled trials—trial 1 (Do CHANGE), which has been registered on www.clinicaltrials.gov (NCT02946281), has provided input for the further development and improvement of the currently described trial 2 (Do CHANGE 2, NCT03178305).

### Study Population

Patients primarily diagnosed with coronary artery disease (CAD) (having experienced a myocardial infarction, percutaneous coronary intervention, angina pectoris and/or coronary artery bypass graft surgery), symptomatic heart failure (HF) (New York Heart Association class I-IV), and patients diagnosed with hypertension (HT) will be included in the study. Hypertension is defined by values ≥140 mmHg of systolic blood pressure or ≥90 mmHg of diastolic blood pressure in two different measurements spaced 1-2 minutes apart and after 3-5 minutes in the sitting position. The values associated with the second measure will be used.

*Inclusion criteria:* Age 18-75 years, diagnosed (primary diagnosis) with CAD, HF or HT, having at least two of the following risk factors: smoking, positive family history, increased cholesterol, diabetes, sedentary lifestyle, and/or psychosocial risk factors. Patients should also have access to the Internet at home, have a smartphone which is compatible with the applications that will be used in the study (and have sufficient knowledge on using a personal computer and smartphone), and speak the countries’ native language.

Additional inclusion criteria for only HF patients include a diagnosis of systolic or diastolic heart failure and presence of HF symptoms (eg, shortness of breath, chest pain, and exhaustion).

*Exclusion criteria:* Significant cognitive impairments (eg, dementia), patients who are on the waiting list for heart transplantation, life expectancy <1 year, life-threatening comorbidities (eg, cancers), with a history of psychiatric illness excluding anxiety and/or depression, patients who do not have access to the Internet or a compatible smartphone, and patients with insufficient knowledge of the local language (Catalonian, Chinese or Dutch). Patients who have participated in the first phase of the Do CHANGE trial will also be excluded.

### Sample Size

A total number of 250 patients will be enrolled in the study at three participating centers (Badalona Serveis Assistencials, Badalona, Spain [N=75]; Buddhist Tzu Chi Dalin General Hospital, Dalin, Taiwan [N=100] and Elisabeth-TweeSteden Hospital, Tilburg, The Netherlands [N=75]). The sample sizes were determined based on the number of patients visiting the outpatient clinic per center.

One hundred and twenty-five patients will be enrolled in the intervention group and 125 in the control group. Considering participation rates in the previously performed Do CHANGE trial and other randomized controlled trials, we expect a significant number (50%) of patients to refuse participation. Hence, we will need to approach 500 patients. As the current trial aims to provide a proof of concept, sample size calculation will not be performed. A total of 250 patients is considered sufficient to meet this purpose.

### Randomization

Patients will be randomized (2:2) to either the intervention group or the control group (ie, usual care). Patients will be randomized using computerized block randomization (stacks of 4). The computer will generate randomization sequences that will be sealed by an independent researcher. Due to the nature of the study, the blinding of the researchers and health care providers is not possible.

### Study Procedure

Patients who fulfill all the inclusion criteria and none of the exclusion criteria will be approached for participation. Due to differences in health care implementation across the three participating countries, the logistics of patient recruitment per site might slightly differ.

### Overall Procedure

The health care professional (cardiologist or cardiac nurse) will inform the patient about the study (orally and in writing). Patients who are willing to participate will be provided with an informed consent and will be given ten days to consider their participation. The research assistant will contact the patients by telephone and schedule a face-to-face appointment if they are willing to participate. During the face-to-face meeting, patients will sign the informed consent (together with the researcher) and will fill in the first set of questionnaires (shown in Table 1).

After completing the questionnaires, they will be randomized to either the intervention or “care as usual” group. Patients randomized to the intervention group will be provided with tools (as described in the “Intervention” section) and instructions on how to use them. Relevant tools and/or applications will be installed on patients’ mobile phone by the research assistant such that the patients will only have to charge the devices. One day after the face-to-face meeting, patients will be contacted by the research assistant by telephone to ensure that all devices are properly charged and that the system is functional. The full intervention (as described below) will be provided for three months. During this period patients will be contacted weekly by the research assistant to evaluate their progress (whether they are compliant or not with the treatment, they will receive feedback about progress).

**Table 1:** Questionnaires.

Questionnaire	No. of items N=177	Construct	Assessment (month)		
			0	3	6
Health Promoting Lifestyle Profile [16]	52	Lifestyle	x	x	x
Do Something Different	45	Behavioral flexibility	x	x	x
World Health Organization quality of life questionnaire [17]	26	Quality of life	x	x	x
Unified Theory of Acceptance and Use of Technology [18]	28	Usability of tools; acceptance of tools; willingness to pay^a^			x
EuroQOL questionnaire [19]	5	Cost-effectiveness	x	x	x
Purpose designed^b^	4	Health care consumption	x	x	x
Patient Health Questionnaire [20]	9	Depression	x	x	x
Generalized Anxiety Disorder scale [21]	7	Anxiety	x	x	x
Distressed Personality scale [22]	14	Type D personality	x	x	x
Client Satisfaction Questionnaire [23]	8	Patient perceived satisfaction^a^			x

^a^These 3 constructs will only be assessed in the intervention group.

^b^Designed by the authors.

**Figure 1:**
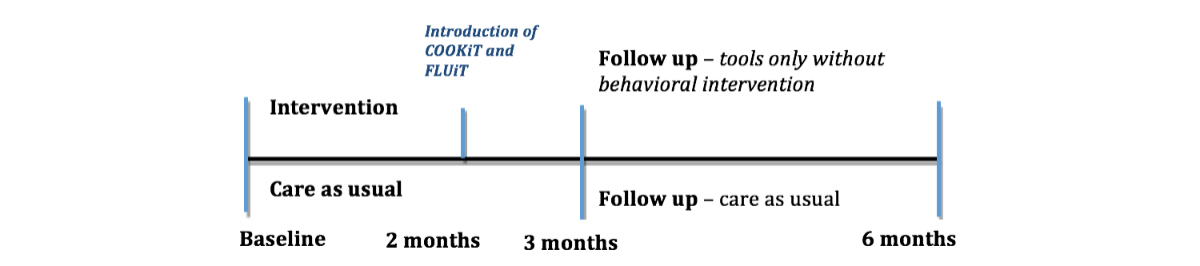
Study procedure.

After the three months period, the behavior change component (the DSD program) will be terminated. Hence, between months 3 and 6, patients in the intervention group will only be using the devices that were provided to them at baseline. In addition, to decrease patient burden, some of the tools and/or components will be introduced two months after baseline measurement (see Figure 1 for a schematic overview of the study procedure).

Patients will be instructed to fill in the first set of questionnaires during their face-to-face visit at the hospital. At 3 and 6 months, the patients will receive a link (by email) to access the follow-up questionnaires online with the instruction to complete them within ten working days. If they do not complete the questionnaires within the given time, patients will receive up to three reminder phone calls.

Patients who have been randomized to the intervention group will also be contacted between months 3 and 6 to participate in a qualitative survey (if possible with their partner), which will assess additional properties of the usability and acceptability of the tools.

### Study Objectives

Primary, secondary and exploratory objectives will be assessed as described below.

#### Primary Objectives

Primary objectives of the trial include: 1) lifestyle (eg, sleep, physical activity, nutrition) change and disease management; 2) enhancement of quality of life; and 3) change in behavioral habits and personal flexibility. The assessment of these objectives will be performed using standardized and validated questionnaires (Table 1). In addition, purpose designed questionnaires will be administered to evaluate changes in objective #3. Furthermore, objective measures (ie, data from devices used by the intervention group) will be employed to evaluate changes in lifestyle variables. However, this will be done only within the intervention group since the “care as usual” group will not receive any devices.

#### Secondary Objectives

Secondary objectives will include: 1) assessment of satisfaction, usability, and acceptance of the intervention (tools); 2) evaluation of the cost-effectiveness of the intervention; and 3) evaluation of changes in health care consumption. Objective #1 will be achieved through validated questionnaires and qualitative interviews with end users and their caregivers (if applicable) and objective #2 will be evaluated using standardized and validated questionnaires only (Table 1). Objective #3 will be assessed using a purpose designed questionnaire.

#### Exploratory Objectives

The current trial will also cover a number of exploratory objectives where the focus will be on 1) identifying subgroups of patients who might benefit the most from this intervention based on their profile (eg, psychological and/or disease profile); 2) evaluating the effects of the intervention on the electrocardiogram (ECG) data; and 3) gaining more insight in patients sleep patterns and physical activity over a prolonged period of time. These objectives will mainly serve the development of our new hypothesis regarding successful lifestyle change and will be tested using latent class analysis (LatentGold 5.0).

### Intervention Versus Care as Usual

#### Intervention

**Figure 2:**
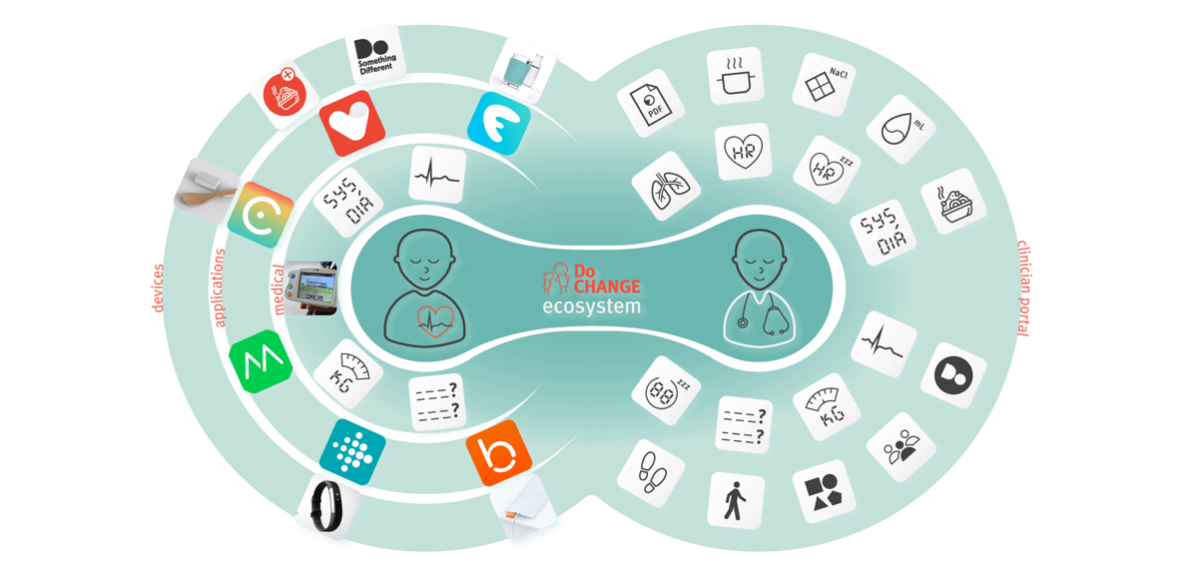
Schematic overview of the Do CHANGE intervention components.

The Do CHANGE intervention consists of different components, which can be used to provide care that meets patients’ needs. All patients included in the intervention group will be provided with the following technology: CarePortal, Moves app, Do Something Different (behavioral program), Beddit, Fitbit, blood pressure monitor, COOKiT, and Vire (the Do CHANGE app). In addition, disease-specific tools will only be provided to those who need them (ie, weight scale and/or FLUiT). Based on patients’ primary diagnosis (HF, CAD, or HT) the tools that might be useful will be recommended by the cardiologist. For example, patients with HF will receive a weight scale, since this can assist with monitoring sudden weigh gain, which might be an indication of deteriorating cardiac function. Figure 2 provides a schematic overview of the intervention components. To decrease patient burden, the COOKiT and FLUiT will be introduced to patients two months postbaseline measurement.

#### Do Something Different

All patients randomized to the intervention group will receive the DSD program, which has previously been developed to change behavioral habits and flexibility [13]. For the current trial, the program has been adapted to cardiac patients’ needs and profiles to meet the lifestyle recommendations of this specific population.

The program challenges patients to step out of their comfort zone by sending behavioral prompts (Do’s) such as “Explore more today instead of going the same old way, take a different route. Look around, spot ten things you wouldn't see on your usual journey.” By breaking the old unhealthy habits, patients are expected to be more flexible and able to change their behavior. The program aims to change behavioral habits, increase flexibility, and subsequently change habits associated with an unhealthy lifestyle and distress, which are both found to be associated with hypertension and cardiovascular risks. “Typical” behavioral risks have been identified and are addressed within the program. To further adapt the program to patients’ needs, all patients, prior to starting the program, will be assessed regarding their daily functioning, distress, and personality such that the Do’s will match their personal (unhealthy) habits and challenge them to change.

After assessing patients’ personality profile, the intervention will be provided for 11 weeks. Patients will receive a total of 32 Do’s messages during this period. Also, 16 ToDo’s will be delivered to the patients based on their current functioning (eg, if a patient is not performing sufficient exercise, based on the Fitbit data, he/she will receive a Do based on that). Patients will receive their Do’s and ToDo’s through the care portal, the Do CHANGE app and via short message service, depending on patients’ preferences.

The intervention group will also receive some devices which will help them to monitor their health behaviors and give them some insight into their daily functioning. These devices will include:

##### CarePortal

The intervention group will also receive a CarePortal (Docobo Ltd), which will be installed at their home. The CarePortal is a clinically certified portable device that will allow the patients to monitor their disease symptoms on a daily basis (ie, by answering a set of predefined questions every day) and send these outputs to a health care professional (cardiologist). The CarePortal will be used to gather ECG data, symptomatic data, blood pressure, and weight on a daily basis. The patient will be able to take the ECG measure at any time. By touching the screen of the CarePortal, the instructions to take the measurement will appear, guiding the patient step by step to take the ECG (which will take 2 minutes). The CarePortal will send the physiological data directly to the cardiologist, who will be able to view them via an online platform and contact the patient if necessary. Also, the patients will see their data (the same data their cardiologist will receive) over a period of 6 months by accessing the online patient portal.

##### Beddit

To objectively log patients’ sleep data and evaluate whether their sleep pattern has changed over time, patients assigned to the intervention group will all receive the Beddit device [16]. Beddit is a certified device to measure sleep, heart rate, and breathing during time spent in bed. The device has been validated [17] and is considered one of the most accurate devices to monitor sleep. For the current trial the Beddit 3 will be used.

##### Fitbit

Patients’ physical activity will be assessed using the Fitbit “Alta HR” [24]. The Fitbit Altra HR is a European Conformity (CE-Marked) activity tracker and can be worn on the wrist. With the Fitbit, patients’ step count, the intensity of physical activity, heart rate, calories burned and distance walked will be assessed. Data from the Fitbit will also be used to initiate ToDo’s.

##### Blood Pressure Monitor

All patients in the intervention group will receive the digital blood pressure monitor, UA-767 Plus, which is a CE-Marked medical device. Patients will be asked to measure their blood pressure on a daily basis and log the blood pressure values through the CarePortal.

##### COOKiT

COOKiT is a smart spatula that can monitor patients’ cooking behavior (through a motion sensor which indicates whether the spatula is used) and measure the salinity—for both sodium and potassium—of the food that is prepared. The COOKiT has been developed within Do CHANGE and will be provided to patients two months postbaseline measurement.

##### Vire (Do CHANGE App)

In order for patients not to feel overwhelmed by the apps and devices that they will have to check every day (if they are interested in their progress), the Vire app (Do CHANGE app) has been developed as the integration point. The Vire app was developed together with end users and health care professionals to provide an overview of the data gathered by all the devices that the patients will be using during the study (eg, Beddit, Fitbit, COOKiT, etc). Through this application, patients will also be able to receive the Do’s from the DSD program. In addition, patients will be asked to take pictures (at least 3 per day) of the food which they have consumed each day (via this app). These images will automatically be sent to the health care professional portal (which is also linked to the CarePortal) such that the health care professional will be able to see what the eating habits of the patients are.

##### Moves App

Moves app is an activity and global positioning system tracking application installed on the mobile phone. The app helps to provide information useful for assessing the behavioral indicators—social opportunity, variety, and activity—used for generating ToDo’s. All patients participating in the intervention will have to install the Moves app on their mobile phone.

#### Additional Devices

Dependent on the primary diagnosis, the patient will decide together with the health care professional which of the following, additional devices they will be using:

##### FLUiT

Particularly for patients with HF the FLUiT will be recommended. FLUiT is a newly developed device in Do CHANGE which can measure fluid intake. FLUiT is a “smart sleeve” which can be wrapped around a cup, bottle or glass, and will gauge the amount of fluid that it contains. It comprises of an accelerometer and a touch sensor to detect when actual drinking occurs and will log the quantity drank. FLUiT will be provided to patients two months postbaseline measures.

##### Weight Scale

Patients with HF will be provided with a weight scale to monitor their weight on a daily basis. For the current trial, the Seca Aura 807 model will be used. Patients will be able to communicate their weight on a daily basis by answering the question on the CarePortal.

Patients will all be monitored weekly via telephone to make sure that they are compliant with the program. During these phone calls, patients will also receive feedback on their eating habits.

### Care as Usual

Patients who are randomized to the comparator group will receive care as usual. There will be no restrictions on this group. Patients in this condition are allowed to seek additional care and also use other tools, which will enhance their disease and well-being, provided that they report this in the purpose designed questionnaires at follow-up.

### Statistical Analyses

Data will be analyzed using SPSS (IBM statistics 22) and LatentGold (Version 5.0) [24,25] statistical package. Continuous and discrete variables will be compared using respectively Students’ t-test and Chi-square test. The Linear Mixed Models (LMM) procedure will be performed to evaluate the treatment effectiveness over time. The LMM procedure is similar to linear regression analyses except that in LMM the dependent variable is measured at multiple time points. These analyses will be adjusted for baseline distress levels. If the interaction effects are not significant, only the main effects will be entered in the final model. To examine which patients might benefit from the intervention based on their profiles, Latent Class Analyses will be performed [26,27]. An alpha of .05 will be used to indicate level of significance.

## Results

The acquisition of trial data described in this paper is expected to be finished during the summer of 2018. The following data analysis and additional publications are expected in the winter of 2018 and spring 2019.

## Discussion

Cardiovascular diseases are the leading cause of death globally, and they pose a significant burden on current health care systems [1,28]. Studies have shown that behavioral factors (eg, physical inactivity, unhealthy diet) account for a large proportion of the disease burden and thus should be addressed (potentially) by health care providers [2]. The Do CHANGE trial aims to address lifestyle behaviors and increase the quality of life of patients with CAD, HF, and HT. By increasing patients’ behavioral flexibility and providing them with supportive devices, patients are expected to break with their unhealthy habits and change their unhealthy lifestyle in a sustainable manner.

According to Pine & Fletcher [13] there are multiple reasons why current behavioral programs have not been successful in changing health behaviors. Firstly, our behavior is only partly guided by willpower, hence, most patients fail to use willpower to prevent habitual behaviors (eg, smoking). Secondly, behavior is often guided by everyday environmental cues which trigger certain response (eg, having a beer triggers smoking). Finally, there is a knowledge-doing gap which is demonstrated by the fact that we often know what is good for our health but we fail to do it [12,13]. People tend to live by their habits, which can be stable over time, and seem not to be able to act in accordance with their knowledge. Hence, within Do CHANGE, we aim to increase awareness and knowledge about unhealthy behaviors and at the same time, by using the DSD technique, disrupt habit chains in daily living. This is expected to boost behavioral flexibility and enhance behavior change [15].

A total of 250 patients will be recruited from three pilot sites (Spain, The Netherlands, and Taiwan) and will be randomized to either the intervention group or care as usual. Patients in the intervention group will receive the DSD program (for three months) and will be using devices that will assist them in behavior change (for 6 months). Patients will be assessed at baseline, and at the third and sixth months.

The current trial might face some challenges due to the international scope and the number of devices that are given to patients. As patient recruitment will take place in Spain, The Netherlands, and Taiwan, cultural differences might affect the outcomes of the trial. Also, due to logistical differences between the pilot sites, patient recruitment will be slightly different per site. Although we do not expect this to have a significant impact on the results, we do consider it as a challenge. Secondly, some patients will receive multiple devices and/or apps that they are expected to use for 6 months. This might be perceived as overwhelming and may potentially lead to lower adherence. However, the current trial implements a behavior change component to the intervention, which aims to increase personal flexibility and enables behavior change. This might lead to more openness for new behaviors (using devices) and thus improve adherence.

Do CHANGE will provide insights into lifestyle changes and the possible mechanism that might drive this change. In addition, it will give valuable information from objective measures about patients’ behavioral patterns which, in turn, could serve as input for future studies that will focus on personalized medicine. This information will also provide input for the development of future ecological momentary interventions (real time interventions) that are focused on providing care to patients whenever and wherever they prefer it.

## Appendix

Multimedia Appendix 1 Peer-review report.

## Abbreviations

CAD: coronary artery disease
CE-Mark: European Conformity mark
Do CHANGE: Do Cardiac Health: Advanced New Generation Ecosystem
DSD: Do Something Different
ECG: electrocardiogram
HF: heart failure
HT: hypertension
LMM: Linear Mixed Models

## References

[1] (2018). Cardiosmart.

[2] Newton JN, Briggs ADM, Murray CJL, Dicker D, Foreman KJ, Wang H, Naghavi M, Forouzanfar MH, Ohno SL, Barber RM, Vos T, Stanaway JD, Schmidt JC, Hughes AJ, Fay DFJ, Ecob R, Gresser C, McKee M, Rutter H, Abubakar I, Ali R, Anderson HR, Banerjee A, Bennett DA, Bernabé Eduardo, Bhui KS, Biryukov SM, Bourne RR, Brayne CEG, Bruce NG, Brugha TS, Burch M, Capewell S, Casey D, Chowdhury R, Coates MM, Cooper C, Critchley JA, Dargan PI, Dherani MK, Elliott P, Ezzati M, Fenton KA, Fraser MS, Fürst Thomas, Greaves F, Green MA, Gunnell DJ, Hannigan BM, Hay RJ, Hay SI, Hemingway H, Larson HJ, Looker KJ, Lunevicius R, Lyons RA, Marcenes W, Mason-Jones AJ, Matthews FE, Moller H, Murdoch ME, Newton CR, Pearce N, Piel FB, Pope D, Rahimi K, Rodriguez A, Scarborough P, Schumacher AE, Shiue I, Smeeth L, Tedstone A, Valabhji J, Williams HC, Wolfe CDA, Woolf AD, Davis ACJ (2015). Changes in health in England, with analysis by English regions and areas of deprivation, 1990-2013: a systematic analysis for the Global Burden of Disease Study 2013. Lancet.

[3] Lloyd-Jones DM, Hong Y, Labarthe D, Mozaffarian D, Appel LJ, Van HL, Greenlund K, Daniels S, Nichol G, Tomaselli GF, Arnett DK, Fonarow GC, Ho PM, Lauer MS, Masoudi FA, Robertson RM, Roger V, Schwamm LH, Sorlie P, Yancy CW, Rosamond WD, American HASPTFC (2010). Defining and setting national goals for cardiovascular health promotion and disease reduction: the American Heart Association's strategic Impact Goal through 2020 and beyond. Circulation.

[4] Roger VL, Go AS, Lloyd-Jones DM, Benjamin EJ, Berry JD, Borden WB, Bravata DM, Dai S, Ford ES, Fox CS, Fullerton HJ, Gillespie C, Hailpern SM, Heit JA, Howard VJ, Kissela BM, Kittner SJ, Lackland DT, Lichtman JH, Lisabeth LD, Makuc DM, Marcus GM, Marelli A, Matchar DB, Moy CS, Mozaffarian D, Mussolino ME, Nichol G, Paynter NP, Soliman EZ, Sorlie PD, Sotoodehnia N, Turan TN, Virani SS, Wong ND, Woo D, Turner MB, American HASCSS (2012). Heart disease and stroke statistics--2012 update: a report from the American Heart Association. Circulation.

[5] Spring B, Ockene JK, Gidding SS, Mozaffarian D, Moore S, Rosal MC, Brown MD, Vafiadis DK, Cohen DL, Burke LE, Lloyd-Jones D, American HABCCOTCOECOLHCFHBPROCN (2013). Better population health through behavior change in adults: a call to action. Circulation.

[6] Hivert M, Arena R, Forman DE, Kris-Etherton PM, McBride PE, Pate RR, Spring B, Trilk J, Van HLV, Kraus WE, American Heart Association Physical Activity Committee of the Council on Lifestyle Cardiometabolic Health; the Behavior Change Committee‚ a joint committee of the Council on Lifestyle Cardiometabolic Health and the Council on EpidemiologyPrevention; the Exercise‚ Cardiac Rehabilitation‚ and Secondary Prevention Committee of the Council on Clinical Cardiology; and the Council on Cardiovascular Stroke Nursing (2016). Medical Training to Achieve Competency in Lifestyle Counseling: An Essential Foundation for Prevention and Treatment of Cardiovascular Diseases and Other Chronic Medical Conditions: A Scientific Statement From the American Heart Association. Circulation.

[7] Lobelo F, Duperly J, Frank E (2009). Physical activity habits of doctors and medical students influence their counselling practices. Br J Sports Med.

[8] Direito A, Carraça E, Rawstorn J, Whittaker R, Maddison R (2017). mHealth Technologies to Influence Physical Activity and Sedentary Behaviors: Behavior Change Techniques, Systematic Review and Meta-Analysis of Randomized Controlled Trials. Ann Behav Med.

[9] Geller K, Lippke S, Nigg CR (2017). Future directions of multiple behavior change research. J Behav Med.

[10] Moller AC, Merchant G, Conroy DE, West R, Hekler E, Kugler KC, Michie S (2017). Applying and advancing behavior change theories and techniques in the context of a digital health revolution: proposals for more effectively realizing untapped potential. J Behav Med.

[11] Habibović M, Burg MM, Pedersen SS (2013). Behavioral interventions in patients with an implantable cardioverter defibrillator: lessons learned and where to go from here?. Pacing Clin Electrophysiol.

[12] Moltu C, Stefansen J, Svisdahl M, Veseth M (2012). Negotiating the coresearcher mandate - service users' experiences of doing collaborative research on mental health. Disabil Rehabil.

[13] Pine K, Fletcher BC (2014). Time to shift brain channels to bring about effective changes in health behaviour. Perspect Public Health.

[14] Baumeister RF, Bratslavsky E, Muraven M, Tice DM (1998). Ego depletion: Is the active self a limited resource?. Journal of Personality and Social Psychology.

[15] Fletcher B(, Hanson J, Page N, Pine K (2011). FIT – Do Something Different. Swiss Journal of Psychology.

[16] Walker S, Sechrist K, Pender N (1995). University of Michigan.

[17] Trompenaars FJ, Masthoff ED, Van HGL, Hodiamont PP, De VJ (2005). Content validity, construct validity, and reliability of the WHOQOL-Bref in a population of Dutch adult psychiatric outpatients. Qual Life Res.

[18] Venkatesh V, Thong J, Xu X (2012). Forthcoming in MIS Quarterly;36(1).

[19] Lamers LM, McDonnell J, Stalmeier PFM, Krabbe PFM, Busschbach JJV (2006). The Dutch tariff: results and arguments for an effective design for national EQ-5D valuation studies. Health Econ.

[20] Kroenke K, Spitzer RL, Williams JB (2001). The PHQ-9: validity of a brief depression severity measure. J Gen Intern Med.

[21] Spitzer RL, Kroenke K, Williams JBW, Löwe B (2006). A brief measure for assessing generalized anxiety disorder: the GAD-7. Arch Intern Med.

[22] Denollet J (2005). DS14: standard assessment of negative affectivity, social inhibition, and Type D personality. Psychosom Med.

[23] Clifford A CSQ Subscales.

[24] (2018). Fitbit Inc.

[25] Paalasmaa J, Toivonen H, Partinen M (2015). Adaptive Heartbeat modeling for beat-to-beat heart rate measurement in ballistocardiograms. IEEE J Biomed Health Inform.

[26] Vermunt J (2003). Applications of latent class analysis in social science research. Lect Notes Artif Int.

[27] Vermunt JK, Magidson J (2003). Latent class models for classification. Computational Statistics & Data Analysis.

[28] (2018). World Health Organization.

