# Supplementary material for: Enhancing Lifestyle Change in Cardiac Patients Through the Do CHANGE System (“Do Cardiac Health: Advanced New Generation Ecosystem”): Randomized Controlled Trial Protocol
Source: JMIR Res Protoc. 2018 Feb 8;7(2):e40. doi: 10.2196/resprot.8406 (PMC5824100; doi:10.2196/resprot.8406)
Supplement: Multimedia Appendix 1 [file resprot_v7i2e40_app1.pdf]

EUROPEAN COMMISSION
Directorate-General for Communications Networks, Content and Technology

Sustainable and Secure Society
Health & Well-Being

CONSOLIDATED REVIEW REPORT

**Grant Agreement (GA) number:**	643735
**Project Acronym:**	Do CHANGE
**Project title:**	Do Cardiac Health: Advanced New Generation Ecosystem
**Type of Action:**	RIA
**Start date of the project:**	01/02/2015 (start date after amendment)
**Duration of the project:**	36
**Name of the representative of the project's coordinator and organisation:**	Ad Van Berlo
**Period covered by the report**:	from 01/02/2015 to 31.01.2016
**Periodic report:**	1st
**Date of submission of the periodic report:**	19/02/2016
**Date of latest version of Annex 1 to the GA (Description of the Action - DoA) against which the assessment is performed**	19/02/2016
**Date of review meeting (if applicable):**	19/02/2016
**Name of external experts assisting in the project assessment (if applicable)**	Ana Vujaklija Brajkovic<br>Hannes Kaufmann<br>Panayotis Vassilakis
**Name of Project Officer drafting the report:**	Stefanos Gouvras

1. Overall assessment

Overall assessment
The project has achieved most of its objectives and milestones for the period with relatively minor deviations. Corrective actions (especially regarding the planned evaluation) will be required.

General comments
The primary objective of Do CHANGE is to develop a health ecosystem for disease management of patients with ischemic heart disease or heart failure. The system incorporates behaviour change methods in conjunction with new mobile tools that can scan the nature of food and fluid intake, monitor behaviour and clinical parameters in normal living situations.<br><br>The project has accomplished all the important tasks of the 1st year but some concerns are remaining with respect to the DoA. The quality of work delivered is rather good although the work of WP4 is not approved and several limitations regarding sensors are identified. Further, the integration of sensors data and Do's into the existing "doc@HOME" Portal has been accomplished. The Project at the end of 1st year presented the necessary low fidelity prototypes ready for validation also with the relevant integration plans. This progress is justifying that the project is on track.<br><br>The work in all WPs was in line with DoA with the exception the work of WP4 which should be further clarified. The work of WP2 & WP3 is planned to follow an annually iterative cyclic process safeguarding that tools and services are refined from concept to final validation and usage. At this stage the user requirements framework is acceptable and only minor modifications are needed to be done within the update of the coming period regarding a more sufficient presentation of the users' requirement list. The work of WP3 is partially accomplished especially for tools that planned to be used for salt intake and food composite scanning. On the contrary the work refers to liquid intake measurement is ahead of schedule. Overall, based on the work already performed, it is expected that the selection of the most preferable tools for salt and water intake, and food composite scanning could be successfully achieved within the coming period.<br><br>The work of WP4 is partially accomplished since one deliverable is not submitted and the work presented is contributing only to the general architecture regarding management of personal medical data. More, parts of the submitted deliverables of this WP found to have common texts with parts of deliverables of TAS3 an FP7 project. Overall, from the work presented it is not evident that WP4 has explored specific requirements of the targeted users of DoCHANGE project. In this sense the deliverables of this WP are rejected.<br><br>The work of WP5 was affected by changes in the work-plan with respect to the original DoA. The deliverables submitted are acceptable for this stage and are in line with the amended DoA.<br><br>The deliverables of WP6 refers to testing methodology in controlled environments is presenting convincingly the work performed. However, a revision of the study design is needed, as discussed and agreed during the review meeting. The revision refers to the increase of patient population in trials. Partners initially suggested recruiting a population of 150 patients (75 intervention, 75 control). Finally was made clear that minimum amount of 300 patients is needed in order the proper volume of patient data to be collected and a sound statistical analysis and clinical validation to be achieved. Note that a proper clinical validation process will safeguard the exploitation potential. It is expected that the existing work of WP6 will be refined after the finalization of tools presented in WP3.<br><br>The work referring to dissemination planning is sufficiently presented. All the stakeholders and the involved groups of the planned dissemination strategy are identified. The means for the promotion of the dissemination strategy are presented with a clear manner. Nevertheless, the dissemination strategy is not providing efficiency metrics in order to quantitative a more convincing ambition for impact especially in terms of patients groups and health professionals. Partners identified all the aspects of the impact potential in terms of a sufficient qualitative analysis. Nevertheless this analysis is limited in terms of quantified goals and the update version is necessary to explore commercially-related impacts in relation with a specific exploitation proposal. The introduced exploitation proposal explored the market potential in a generic manner since there is still insufficient view for the final specifications of the tools of WP3. However, it is evident that DoCHANGE ecosystem and its elements are establishing a clear vision for the promotion of ambient iHealth services to the targeted users.

The management scheme has established several efficient tools for plan and control of the work and the steering processes appear to be productive.<br><br>The work that has been carried out is mostly complying with the DoA plan for delivering innovative products. It is evident that partners are aware of the state of the art and more they have a detailed plan for achieving to develop operational prototypes. The innovations already identified can strengthen the competitiveness of the related SMEs and organizations. Considering these innovations and the evolving market of iHealth, it is understood that the provided exploitation proposal needs to be updated providing a convincing business case that should be proper for increase the competiveness of involved SMEs.<br><br>The beneficiaries in general are contributing in line with the work in the DoA. Nevertheless, partner "SYNERGETICS", which mostly is involved in work of WP4, didn't provide data for justifying its contribution. In addition the contribution of SYNERGETICS is questionable due mostly to the fact that the work performed in WP4 is addressing only generic level requirements not necessarily within the context of DoCHANGE targeted users and was not accepted (see the relevant comments above). These overall are making evident that SYNERGETICS is not justifying contribution in terms of work committed in the DoA.<br><br>In general the use of resources was more or less justified by all partners with the exception of Partner "SYNERGETICS". In line with this the effort claimed by this partner is not documented and it is not possible to justify the requested payment.
**Recommendations concerning the period under review**
Partners followed the recommendations of the interim review of the 1st year.
**Recommendations concerning future work**
R1.1: The presented work of WP4 is not clearly addressing specific requirements of the targeted users of DoCHANGE ecosystem. The submitted deliverables need to be rewritten and restructured for complying with ecosystem scope. It is suggested this work to be resubmitted in the coming period.<br><br>R1.2: Partners have proposed an approach for trials based on a rather limited population of patients. However, during the review meeting was agreed that for the trial process a minimum amount of 300 patients will be recruited. This population can support a sound clinical validation which is necessary prerequisite for achieving exploitation potential.<br><br>R1.3: The Dissemination Strategy it is expected to be reinforced during the coming period. Partners are competent to prepare and implement a successful and proper dissemination activity. Within this context it is suggested to prepare indices for evaluation of the dissemination efficiency per involved group of stakeholders and targeted users. These metrics can be encompassed as a new section in the updated dissemination plan of the coming period.<br><br>R1.4: Partners following the recommendation of the interim review of the 1st year prepared an early impact & exploitation proposal. A new updated exploitation proposal is needed the soonest possible within the coming period. This update is recommended to encompass (a) Plans for the promotion of ecosystem, (b) Marketing tactics addressing the issue of loyalty of targeted users, (c) Models for revenue per users' segment, (d) Competition analysis.<br><br>R1.5: From the work of WP3 it is evident that innovative devices and services will be developed within the project lifetime. It is clear that partners need to explore properly the issues of IPR and the potential of patented know-how. This work can be a special section of the updated impact and exploitation proposal of the coming period.<br><br>R1.6: The project in the coming 6 months has to achieve critical technological goals. In addition, the restructuring of the work of WP4 it is necessary to be finalised the soonest possible. Considering these, it is recommended an interim review that will access the progress of the next 6-8 months. It is recommending this interim review to access the new work of the WP4, the progress of work in WP3 and finally the progress in dissemination and exploitation.

2. Objectives and Workplan

**Is the progress reported in line with objectives and work plan as specified in the DoA? If**	Yes

**there are significant deviations, please comment.**	

The project has accomplished all the important tasks of the 1st year and minor concerns are remaining with respect to the DoA. The quality of work delivered is rather good although one deliverable is missing and several limitations regarding sensors are identified. Further, the integration of sensors data and Do's into the existing "doc@HOME" Portal has been accomplished. Last but not least the first API's with the total Do CHANGE infrastructure have been generated and built. Thus the project at the end of 1st year presented the necessary low fidelity prototypes ready for validation also with the relevant integration plans. This progress is justifying that the project is on track.

WP 1: Project management

The Project Management scheme is overall operational and efficient. For the support of the project management procedures a collaborative tool was installed, the so-called Teamwork Portal. It is expected that with this tool the communication and the interactivity among partners will be further promoted. In addition this tool provides a dashboard for continuing overview of the Project material. The work of D1.2 referring to Management handbook establishes convincing quality procedures and it is also presenting a sufficient risk control plan. The Ethical & Legal manual which was delivered with the D1.3 is encompassing sufficiently presentation of the ethical regulation in countries where trials will be conducted; in this sense the proposed manual can address issues that could be raised in processes of trial design and implementation. An Ethical Board has been set-up to ensure that the activities carried out in the project by the different pilots abide to national and EC ethical rules. The first periodic review, the D1.4, is presenting sufficiently and with a rather comprehensive manner the work progress of the project and further summarizes the current state.

WP 2: User needs and co-design of tools + services

The work of WP2 will follow an iterative, cyclic process with three cycles of one year each. This process safeguards that tools and services are refined from concept to final validation and usage. Within this context the D2.1a is presenting technical and non-technical requirements regarding the first stage user needs and co-design of tools. This first stage analysis is determining clearly the context per topic; in this sense the requirements' analysis is acceptable. The work of D2.2 is determining Personas and use cases addressing the several issues related with the understanding of the patient behaviour. Although that overall this work is acceptable and has good quality fails to present sufficient presentations of requirements in chapter 10; the presentation of requirements is synoptic and generic. The recommendation from the interim review concerning the number of interviewed clinicians and inclusion criteria including patients' age are adopted.

WP 3: Tools development

The work of WP3 will follow, like WP2, a cyclic and incremental process in implementing and developing the tools for salt and water intake, and food composite scanning. All three tasks started in the reported period. The D3.1 summarizes findings in all three areas in a very clear way and presents results of experiments that evaluate the limits and possibilities of existing and new sensor types, while D3.2 was a convincing demonstration of the current state of the 3 sensors at the review meeting. The work on the salt measuring spoon is innovative and explores the possibilities and practical limits of impedance spectroscopy under different temperatures. The spoon will include additional sensors to analyse cooking behaviour. Overall, the work refers to salinity measurement fails to propose a most preferable salinity sensor although several alternatives were evaluated; the work is extensive in terms of technical tests and specifications and it is expected that within coming period this work will conclude with the preferable sensor device. The liquid measurement sensor work is sufficiently presented. The initial concept of a submersible artifact is abandoned and a sleeve like concept is proposed. Laboratory tests are performed to explore capacitive level sensing techniques. Low-fidelity prototypes are created to realise a first proof of concept and a set of 10 mid-fidelity prototypes were delivered for a study with 40 renal patients and further improvement. In this sense the liquid volume measuring sleeve has made good progress and is ahead of schedule. The food composite scanning tool has been also explored extensively. The work performed is encompassing experiments in controlled laboratory environment for computer vision technique and development of a multipoint spectrometer sensor. Both techniques have several limitations that need to be addressed. It is expected that the final solution will combine the computer vision approach with the spectrometry approach. Nevertheless, a low cost spectrometric sensor might not be available within the project lifetime. The presentation for work planed is sufficient and convincing.

WP 4: Data collection & analytics

A fundamental concept in Do CHANGE is the fact that access to personal medical data will be managed by the individual. All deliverables of WP4 are not specific to DoCHANGE but describe various aspects on a high level. The D4.1.1 introduces a high level architecture of the DoCHANGE ecosystem regarding management of personal medical data. Nevertheless, this document contains wrong references to the TAS3 project (e.g., the table of contents

includes "chapter 7-CONCLUSION: TAS3 IS SECURE AND TRUSTWORTHY, but page 76 states "Conclusion: DO CHANGE is Secure and Trustworthy"). After an online search, it was found that https://zxid.org/tas3/tas3-arch-7tas3arch-ConclusionTAS3isSecureandTrustworthy.html is very similar to the conclusion of D4.1.1. Further, on the website https://zxidp.org/tas3/ - which contains some deliverables of the TAS³ FP7 project – it was identified that D2.1 of the TAS3 project has an extended common text with D4.1.1 (the common text refers to the first 78 pages of the TAS3 D2.1). In addition, the text in "https://zxidp.org/tas3/tas3-biz-model.pdf" (pages 5-29) is also existing in D4.2.1 of DoCHANGE, which presents the trust assurance framework (e.g., the pages 35-50 of D4.2.1 have identical texts with pages 5-29 of the above mentioned TAS3 document). The full text of D2.1 of TAS3 can be found at the following links "https://zxidp.org/tas3/TAS3_D2p1_Arch-v23.pdf". D4.5.2 presents an API management framework that is mainly interesting for larger projects, for the inclusion of new developers and for future extensions. It is not clear why this framework was developed for Do CHANGE or why it is needed in DoCHANGE ecosystem. Thus, the usefulness of API management framework as a subpart is not justified within the DoCHANGE ecosystem since users will not need to use this module. Two demonstrations were given at the review meeting, corresponding to two deliverables, about various aspects of the framework. A major drawback in the WP4 is that the D4.3.1 was not finally delivered for review. Overall, the work of this WP is not accepted and it is necessary to be clarified which part of the presented work has been originally prepared for the DoCHANGE ecosystem. Although the methodology for the management of the personal data by individuals can be directly associated with health personal data, the work should be more focused to the requirements of the users of the DoCHANGE ecosystem.

WP 5: System integration

The objectives of this WP are to integrate and implement the various services and data sources into the Do CHANGE ecosystem and further to implement all types of technical testing. The planned tasks for the reporting period have been achieved and all the foreseen deliverables have been achieved. The D5.1.2 reports on the acceptance testing carried out on completion of the integration of the Baseline functionality of the DSD System with the Docobo doc@HOME System within the context of the Do CHANGE Eco-System. D5.3.1 specifies the functions required for the Collection of Data from the various Do CHANGE Sensors and taking it into the Do CHANGE Eco-System with a particular focus on the requirements for preparing an initial demonstration system for review and marketing purposes and the implementation of the functions specified in D5.3.1. Both reports are sufficient. Since the specifications of the measuring spoon and the spectrometry device are unclear yet, specific functional requirements for these will have to be updated later. The D5.1.1 and D5.3.2 are demonstrators referring to the basic functionality of registering users and collecting their measurements. Both were demonstrated successfully. Partners recognized that they will need to put additional effort in integrating the DoCHANGE ecosystem in different countries due to the differences of the local medical regulations. Additional effort it is also expected by the increase of the patient population in Pilot Sites due to the need to collect the proper volume of patient data that will support a sound statistical analysis and clinical validation.

WP 6: Validation and evaluation of tools and services

The WP6 is the biggest of the project since the WP effort amounts to 120 PMs and encompassing the evaluation framework, deployment planning, ethical approval, recruitment and user mentoring, the operation support and piloting. The D6.1 has a good quality and presents comprehensively the testing methodology in a controlled environment. In the appendices it summarizes the number of tests that have been done so far. The results seem well suited for scientific publication. The D6.2 refers to the first version of the Do CHANGE evaluation framework; this work clearly identifies all the issued regarding the evaluation framework. The methodology and the study protocol are clear. However, a revision of the study design is needed, as discussed and agreed during the review meeting. The revision refers to the increase of patient population in trials. Partners initially suggested recruiting a population of 150 patients (75 intervention, 75 control). Finally was made clear that minimum amount of 300 patients is needed in order the proper volume of patient data to be collected and a sound statistical analysis and clinical validation to be achieved. For the coming period partners will adopt Model for ASessment of mHealth (MASH) which is based on the Model for ASsessment of Telemedicine (MAST) for measuring the impact of the Do CHANGE ecosystem in terms of the social, economic and health indicators. The revised evaluation framework is expected to be presented in D6.7.

WP 7: Dissemination and exploitation

The work of D7.1 referring to dissemination planning is sufficiently presented. All the stakeholders and the involved groups of the planned dissemination strategy are identified. Partners explored global burden of diseases, found research evidence that suggests the need for an increased emphasis on modifiable behavioural risk factors in non-communicable disease generally and it's management, especially in an increasingly aging population. The means for the promotion of the dissemination strategy are presented with a clear manner. Nevertheless, the dissemination strategy is not providing efficiency metrics in order to quantitative a more convincing ambition for impact especially

in terms of patients groups and health professionals. In addition assuming the progress of WP3 work, it is expected the dissemination strategy to determine a more specific potential regarding scientific publications. The advisory board has been established and its composition appears to be balanced in terms of scientific areas.<br><br>The D7.3.1 identifies all the aspects of the impact potential. The impact potential has been explored sufficiently for academic, research and societal issues but in qualitative manner. The general commercially-related impacts are presented but are not linked with the specific exploitation proposal. In addition, the exploitation proposal that was introduced in D7.3.1 explored the market potential in a generic manner. However, it is understood that a more precise business case was not easy to be provided since there are still insufficient view for the final specifications of the tools of WP3. The proposition for the increased value of the DoCHANGE ecosystem and elements to individual patients is convincingly form a vision for the promotion of the tools and services of the Project. The reinforcement of patients through DoCHANGE ecosystem and the ambitious technological tools of WP3 could easily support a successful and viable exploitation proposal. Partners are competent to develop a clear and sound exploitation proposal within the coming period; it is necessary, the update of the exploitation plan to use quantitative market data and to propose a detailed business case.	
**Are the objectives of the project still scientific and /or technological relevant?**	Yes
The objectives are still fully relevant and achievable within the time and resources available to the project. Approach and methodology continue to be relevant. However, the difficulties of partners to develop operable prototypes for salinity measurement and food composite scanning will likely cause delays affecting project's scientific and technological ambition.	
**Are the critical implementation risks and mitigation actions described in the DoA still relevant?**	Yes
The risks are described sufficiently in the DoA, Moreover some new risks have been identified in the periodic report according to the current state of the project. Risks mitigation is provided.	
**Have the pilots/study cases started to showcase innovative results as described in the DoA?**<br>***[Option if applicable]***	
Not applicable yet.	
**Have the ethics specific deliverables and/or requirements due for the current period been properly addressed and approved?**<br>***[Option if applicable]***	Yes
An ethics board has been established. The work for ethical approvals has been planned for coming period and after the finalisation of the technological tools.	
**Have the comments and recommendations from previous assessments been taken into account?**<br>***[Option if applicable]***	Yes
The partners adopted all recommendations that were given at the interim review.	

3. Impact

**Does the work carried out follow the plan detailed in the DoA to deliver innovation to the markets in order to strength the competitiveness and grow of related companies? Give information on the relevant innovation activities carried out (prototypes, testing activities, clinical trials) and/or new product, process or method (to be) launched to the market, if any.**	Yes
The work that has been carried out is mostly complying with the DoA plan for delivering innovative products. Partners are involved in several cases of developing new proper techniques facilitating the ecosystem targeted services. It is evident that partners are aware of the state of the art and more they have a detailed plan for achieving to develop the necessary operational prototypes. The innovative features of devices and tools have been sufficiently presented. The prototypes for the liquid measuring sleeve as well as for the spoon are very promising. The ecosystem as a whole is promoting ambient i-services for patients.	

These innovations can strengthen the competitiveness of the related SMEs and organizations. Considering the innovation identified and the evolving market of "iHealth", it is understood that the provided exploitation proposal needs to be updated providing a convincing business case that should be proper for strengthen the involved SMEs. An interesting part of this business case should be the market promotion and exploitation of services referring to behavioural change of patients.	
**Does the work carried out follow the plan detailed in the DoA to get results contributing to the expected impacts detailed in the relevant Work Programme and to any other environmental and social important impacts?**	Yes
The work carried out follows the plan detailed in the DoA. All the existing demos of early prototypes planned for this stage were finally successful. The project has identified and presented clearly societal impacts. This presentation needs to be updated in the coming period having reference to the patients' segments that could be assisted directly or indirectly to change behaviour.	
**Does the work carried out will have an impact on SMEs?**	Partially
The innovation level of the expected prototypes is significant and documenting an evident competitive advantage. Nevertheless, partners provided an early exploitation proposal which does not provide clear evidences but only expectations that the involved SMEs and organizations will gain increase of competiveness.	

4. Implementation

**Has the project been efficiently and effectively managed? Is the management of the project in line with the obligations of beneficiaries (including ethics and security requirements, risk and innovation management)?**	Yes
The management of the project has established several tools for plan and control of work (specific comments have been presented in section 2 above). The steering processes appear to be productive. Overall, the management of the project is very good. The coordinator efficiently and effectively manages the project. Nevertheless, partner "SYNERGETICS" didn't provide documentation for effort spent (see section 5.2.8 of D1.4, pages 58, 59) and this is an indication that "SYNERGETICS" might not be complying with the coordination rules. Apart of this, it is evident that the management of the project is in line with the obligations of beneficiaries.	
**Is the contribution of each beneficiary in line with the work committed in the DoA?**	Yes
The beneficiaries in general are contributing in line with the work in the DoA. Nevertheless, as mentioned in the paragraph above, partner "SYNERGETICS" didn't provided data for understanding its contribution. In addition the work performed by "SYNERGETICS" in WP4 is not clear that addresses specific requirements of targeted users. The provided deliverables are addressing in general the issue of personal data management by individuals. In addition one deliverable was not submitted according to the plan. Overall, it is clear that "SYNERGETICS" contribution is not evident in terms of work committed in the DoA.	
**Have the beneficiaries disseminated project results (foreground) in scientific publications as planned in the DoA, including the deposition of publications in open access repositories? Has the dissemination plan been updated?**	Yes
The dissemination plan is expected to be deployed in the coming period. The presented plan is updated regarding the version introduced in the interim report; in this updated version all the critical aspects of a proper dissemination plan have successfully identified. Note that one scientific publication has been published so far (note that beneficiaries are expecting 12-15 publications during the lifetime of the project).	
**Have the beneficiaries disseminated and communicated project activities and results by other means than scientific publications (social media, press-release, the project web site, video/film…) as planned in the DoA?**	Yes
The consortium has produced material for raise awareness of the involved communities (videos, brochures, etc). The produced material is well designed and is possible to raise interest of several stakeholders and	

especially of targeted users. Traditional dissemination activities through web have been undertaken. So far the campaign through web & social media attracted a good number of visitors. Nevertheless it is expected that within the coming period all the dissemination activities need to be reinforced.	
**Has the plan for exploitation of results, in particular with regards intellectual property rights, been appropriately planned and executed, as described in the DoA? Has the exploitation plan been updated?**	Partially
The existing documentation is encompassing only vague information regarding the intellectual property that could be developed within the project lifetime. This aspect needs to be explored extensively considering especially the expecting contribution of the project in terms of innovation.	

5. Resources

**Are the resources used in the relevant period connected with the project as described in the DoA and are necessary to achieve its objectives? Have been they used in a manner consistent with the principle of sound financial management, in particular regarding economy, efficiency and effectiveness?**	Yes
Overall, the use of resources was not exceeding the total amount planned for the 1st year. In addition the use of resources was more or less justified by all partners with the exception of Partner "SYNERGETICS". "SYNERGETICS" didn't provide any reference for effort and resources spent. In section 5.2.8 of D1.4 (pages 58 and 59) the data in the table are totally missing. Considering that the work of SYNERGETICS delivered in WP4 addresses mostly generic aspects for handling personal data by individuals and the D4.3.1 was not delivered for review, it is understood that "SYNERGETICS" effort for the 1st year is not reflected the claimed amount. In line with this, the provided documentation and reports are not possible to justify the requested payment for "SYNERGETICS".	

Annex 1 – List of deliverables

Del. no.	Deliverable name	Status	Comments
D1.1	Project Presentation and Project Description Leaflet	Accepted	
D1.2	Management Handbook, Quality and Risk Control Plan	Accepted	
D1.3	Ethical and Legal Manual	Accepted	
D1.4	First Periodic Report	Accepted	
D2.1	Detailed specifications of tools and services	Accepted	
D2.2	First-cycle completion report including integrated use cases	Accepted	
D3.1	Explorations of sensor technology	Accepted	
D3.2	First prototypes of all 3 devices (low fidelity level) with integrated sensors	Accepted	Good demonstration of all 3 devices.
D4.1.1	Overall Do CHANGE ecosystem infrastructure architecture, including PDS and ecosystem component framework – Version 1	Rejected	D4.1.1 is rejected (see explanation in Section 2, WP4).
D4.2.1	The end2end trust assurance framework software – Version 1	Rejected	D4.2.1 is rejected (see explanation in Section 2, WP4).
D4.2.2	The end2end trust assurance framework	Accepted	This was a demonstrator and was presented.
D4.3.1	Do CHANGE semantic Infostructure including data conversion and data flows	Not Submitted	

D4.5.1	The Do Change Application Programming Interfaces Software – Version 1	Accepted	This demonstrator was well presented.
D4.5.2	The Do Change Application Programming Interfaces – Version 1	Accepted	
D5.1.1	Integrated DSD baseline functionality with doc@HOME	Accepted	This deliverable was a demonstrator. The basic functionality of registering users and collecting their measurements (D5.3.2) was demonstrated successfully.
D5.1.2	Report of the DSD baseline functionality integration with doc@HOME	Accepted	
D5.3.1	Functional Specification for the Eco- System sensor data collection and storage Application (Part 1 Sensor Data Collection)	Accepted	
D5.3.2	Do CHANGE Sensor Data Collection Demonstration	Accepted	Successfully demonstrated.
D6.1	Testing methodology in controlled environments	Accepted	
D6.2	First version of the Do CHANGE evaluation framework	Accepted	A revision of the evaluation framework, especially of the study design is needed, as discussed and agreed during the review meeting. (see R1.2 in section "Recommendations concerning future work")
D7.1	Dissemination and networking plan	Accepted	
D7.3.1	First exploration on exploitation and impact analyses of the Do CHANGE ecosystem	Accepted	Accepted under the condition that considerable effort will be put into the updated exploitation plan. It would be best to submit am updated draft of the document (D7.3.2) in the coming period (see R1.4 & R1.6, in section "Recommendations concerning future work")

Annex 2 – List of milestones

Milestone no.	Milestone title	Achieved	Comments
MS1	End of first cycle	Yes	
